# Metastatic pulmonary calcification: contribution of imaging to noninvasive diagnosis

**DOI:** 10.1590/0100-3984.2017.50.5e2

**Published:** 2017

**Authors:** Pedro Paulo Teixeira e Silva Torres

**Affiliations:** 1 MD, Radiologist, Multimagem Diagnósticos, Goiânia, GO, Brazil. E-mail: pedroptstorres@gmail.com.

Metabolic pulmonary diseases comprise a heterogeneous and rare group of diseases, each with their own characteristics but with a common origin (metabolic or biochemical dysfunction); diseases in this group can be localized, affecting only the lungs, or systemic, affecting the lungs together with other organ systems^(1,2)^. One such disease is metastatic pulmonary calcification (MPC), which is caused by disturbances in the calcium-phosphorus metabolism and results in abnormal calcium deposits in normal lung tissue^(3,4)^.

Although MPC is a common diagnosis at autopsy, antemortem diagnosis is uncommon and much of the information about the disease is found in isolated case reports^(4-6)^. In the previous issue of Radiologia Brasileira, Belém et al.^(7)^ published an interesting retrospective descriptive analysis of the patterns of MPC seen on chest tomography. The sizeable sample of 23 patients, resulting from a multicenter collaboration, made it the largest study of the disease to date. The quality of the sampling was also excellent, histopathological confirmation having been obtained in the vast majority of cases. The number of cases included allowed the spectrum of known tomography presentations of the disease to be expanded. In some of the patients, the authors identified reticular opacities with calcified micronodules, which have not previously been reported in MPC^(7)^.

Corroborating the findings of previous studies, Belém et al.^(7)^ demonstrated a clear association between MPC and chronic renal failure, highlighting the strong correlation of the latter condition with cardiovascular events and the greater susceptibility to infections in immunocompromised individuals^(1-4,7,8)^. The Belém et al.^(7)^ finding of ground glass opacities—the most common pattern in their sample—broadens the range of differential diagnoses in this group of patients, including congestive conditions and infections^(9)^. After ground glass opacities, the most common findings were dense consolidations and calcified micronodules, which also involve multiple differential diagnoses, including other metabolic conditions, pneumoconiosis, and drug (amiodarone) toxicity^(10)^. Therefore, knowledge of the tomography patterns of MPC, as described in the article, is useful not only for informing the diagnosis but also for excluding other diagnoses, aspects that could have an impact on patient management.

The magnetic resonance imaging patterns of MPC have also been described^(11)^. In the case series conducted by Hochhegger et al.^(11)^, T1-weighted sequences revealed that lung lesions related to MPC produced signals that were hyperintense in comparison with those of muscle tissue, an aspect due to the specific characteristics of the calcium crystals in the MPC lesions and similar to some patterns seen in brain calcification^(12,13)^. Notable among other noninvasive diagnostic imaging techniques is technetium-99m methylene diphosphonate scintigraphy, which is considered more specific for the diagnosis of MPC and capable of detecting earlier manifestations of the disease^(3)^. In pulmonary involvement, radiopharmaceutical uptake occurs symmetrically, and its deposition can also be observed in the gastric wall, as well as in a variable manner in the renal parenchyma^(3)^.

Although lung biopsy is a valuable tool for the diagnosis of various pulmonary conditions, it is not risk-free and its cost-benefit ratio should be carefully studied by the multidisciplinary team before it is performed^(14)^. Recent studies evaluating complications of lung biopsies at referral centers have reported mortality rates ranging from 1.7% to 3.9%, reaching up to 16% in patients undergoing non-elective biopsy, which underscores the fact that the presence of comorbidities contributes to reducing survival among such patients^(15,16)^. Therefore, studies of diffuse lung diseases with a focus on noninvasive clinical and imaging-based diagnostic techniques, such as that conducted by Belém et al.^(7)^, should be encouraged.

Diffuse lung diseases are often a challenge for the multidisciplinary team, and diagnostic imaging plays a decisive role in many situations. The article authored by Belém et al.^(7)^ makes a significant contribution to the tomography-based diagnosis of and noninvasive approach to MPC, highlighting the valuable role that the radiologist plays in the diagnosis of this condition.
